# Extreme Leukocytosis and Gangrenous Cholecystitis Associated with Cytoreductive Surgery and HIPEC-Treated Mucinos Ovary Cancer: Case Report and Literature Review

**DOI:** 10.3390/clinpract13050102

**Published:** 2023-09-18

**Authors:** Stojan Latincic, Maja Pavlov, Jovica Vasiljevic, Dragan Vasin, Maja Dimic-Cumic, Marjan Micev, Milena Papovic, Miljan Doskovic, Stefan Bugarin, Stefan Milosevic, Dragutin Kecmanovic

**Affiliations:** 1 Clinic for Digestive Surgery—First Surgical Clinic, University Clinical Centre of Belgrade, Koste Todorovica 6, 11000 Belgrade, Serbia; stojan.latincic@gmail.com (S.L.); majapavlov1966@gmail.com (M.P.); majacumic@gmail.com (M.D.-C.); micevm@gmail.com (M.M.); mina.papovic@yahoo.com (M.P.); doskovic.miljanue@gmail.com (M.D.); stefanbugarin94@gmail.com (S.B.); milosevic.stefan92@gmail.com (S.M.); kecmanovicdragutin@gmail.com (D.K.); 2 Emergency Department, University Clinical Centre of Serbia, Pasterova 2, 11000 Begrade, Serbia; draganvasin@gmail.com

**Keywords:** mucinous ovary cancer, extreme leukocytosis, gangrenous cholecystitis, COVID-19, hypercalcemia, cytoreduction, HIPEC

## Abstract

Mucinous ovarian cancer occurs sporadically, with a frequency of approximately 3–5% among all subtypes of ovarian cancer. Extreme leukocytosis >40,000 and 50,000 has been described in most solid tumors and is associated with a poor prognosis, although there is a lack of literal data of its occurrence after cytoreductive surgery and HIPEC in the treatment of advanced mucinous ovarian cancer. There is higher risk of the occurrence of cholecystitis in oncology patients compared to the general population, although there is no formal evidence for this, and the association with ovarian cancer is accompanied by a relative risk of 1.38. Hypercalcemia-hyperleukocytosis is a syndrome associated with head and neck cancers, although, to our knowledge, it has not been described in mucinous ovarian cancer, especially after cytoreductive surgery and HIPEC.

## 1. Introduction

Ovarian cancer (OC) is the second most common gynecologic malignancy and has the highest mortality rate [1]. While high-grade serous OC (HGSOC) is the most common histological subtype of this condition, mucinous ovarian cancer (MOC) occurs sporadically, with an incidence of approximately 3–5% among all ovarian cancer subtypes [2,3]. MOC is the most common histologic subtype in patients younger than 40 years [3]. Known risk factors for HGSOC such as infertility, early menarche, late menopause, non-breastfeeding, and BRCA mutation are not associated with MOC. The only possible risk factor for MOC is smoking [3,4].

While HGSOC is most commonly diagnosed at an advanced stage, MOC is diagnosed at stage 1 in 80% of cases. The prognosis for MOC is better in the early stage and worse in the advanced stage compared to HGSOC, where the prognosis is related to the response to platinum-based chemotherapy [3].

Approximately 80% of mucinous ovarian carcinomas are metastatic, while 80% of primary tumors are stage 1. The most common origin of the metastasis of primary tumors to the ovary is the gastrointestinal tract, being featured in 45% of patients, followed by the pancreas in 20%, cervix and endometrium in 18%, and breasts in 8% [3,5,6]. Tumors larger than 10 cm and localized unilaterally are the primary tumors in 82% of cases of MOC, while unilateral tumors smaller than 10 cm are metastatic tumors in 87% of cases [3]. Bilateral tumors smaller than 10 cm are metastatic in 92% of cases, while bilateral tumors larger than 10 cm are metastatic in 95% of cases [3,7]. Thus, because it has similarities with other mucosal carcinomas, especially colorectal carcinomas, it is necessary to carefully diagnose the primary tumor MOC using pathohistological analysis [3].

The incidence of cholecystitis in cancer patients is higher compared with the general population, although there is no formal evidence of this [8]. There are several reasons for the increased risk. First, gallbladder obstruction occurs in several types of malignancies, and metastases to the liver occur in 30–50% of patients that died of cancer [8,9]. Other causes are malnutrition and rapid weight loss, and about 10–20% of patients with rapid weight loss develop cholelithiasis [8,10]. The third cause is a weaker immune response and the disruption of infection barriers, making oncology patients vulnerable to infections. Finally, several cancers have similar risk factors to cholecystitis (high-fat diet, obesity, smoking, alcohol abuse, liver cirrhosis, steroid hormone use, and older age) [8]. The relative risk of developing cholecystitis during the first 6 months of follow-up was higher in oncology patients (1.95 vs. 1.23) in the male population, with the highest risk occurring in the population aged 61–70 years (RR 2.78) [8]. The relative risk of cholecystitis was highest in patients with pancreatic and colon cancer (9.44 vs. 4.98 95% CI), while there was only one case among 1204 patients with ovarian cancer (RR 1.38 95% CI) [8].

Extreme leukocytosis >40 and 50 × 10^9^/L (4.5–10 × 10/L) has been described within most solid tumors and is associated with poor prognosis [11]. The frequency of its occurrence has not been clearly defined; so far, there are some small case series describing a frequency of 1–4% in non-hematologic patients. Extreme leukocytosis presents a diagnostic dilemma, it is necessary to eliminate causes such as infection, new-onset hematologic malignancy, the use of corticosteroids or hematopoietic growth factors, severe bleeding, or bone metastases with necrosis [11,12]. A paraneoplastic leukemoid reaction (PLR) must be excluded, although PLRs are not yet well defined. A PLR is characterized by the patient usually being clinically stable despite metastatic cancer or high tumor stage. The significance of the paraneoplastic leukemoid reaction in a patient with a solid tumor and pulmonary metastases is not clearly defined as PLR may be only a sign of advanced disease stage. Previous studies suggest a poor prognosis in patients with PLR and a fatal outcome 12 weeks after observing the initial extreme leukocyte count [11]. Extreme leukocytosis in a patient with a solid tumor is rarely associated with infection; PLR must be considered in extreme cases of leukocytosis, especially in patients who are clinically stable and have an advanced solid tumor.

The gold standard in the surgical management of epithelial ovarian cancer, including MOC, is staging surgery in the early stage and cytoreductive surgery in the advanced stage. Staging surgery includes peritoneal lavage for cytologic analysis, hysterectomy with bilateral adnexectomy, pelvic and para-aortic lymphadenectomy, omentectomy, and multiple peritoneal biopsies [3]. Cytoreductive surgery includes the removal of macroscopically visible disease and reduces its presence to the microscopic level. The role of HIPEC in the treatment of epithelial ovarian cancer has increased significantly in recent years. Two meta-analyzes demonstrated a significant survival benefit in epithelial ovarian cancer treated with HIPEC at primary and recurrent stages [3,13,14]. However, a large multicenter study of rare ovarian cancers showed no advantage of using cytoreductive surgery and HIPEC in MOC [15]. Given the similarity between colorectal pseudomyxoma peritoneum in nature and biology, the use of HIPEC is considered to be a reasonable option in the treatment of MOC. The issue of which cytostatic agents should be used for colorectal or gynecologic malignancy is still controversial. Because of the low frequency of MOC, it is difficult to obtain specific data on the association between HIPEC and MOC, although data from studies for ovarian and colorectal epithelial cancers can be used [3].

## 2. Case Report

**Figure 1:**
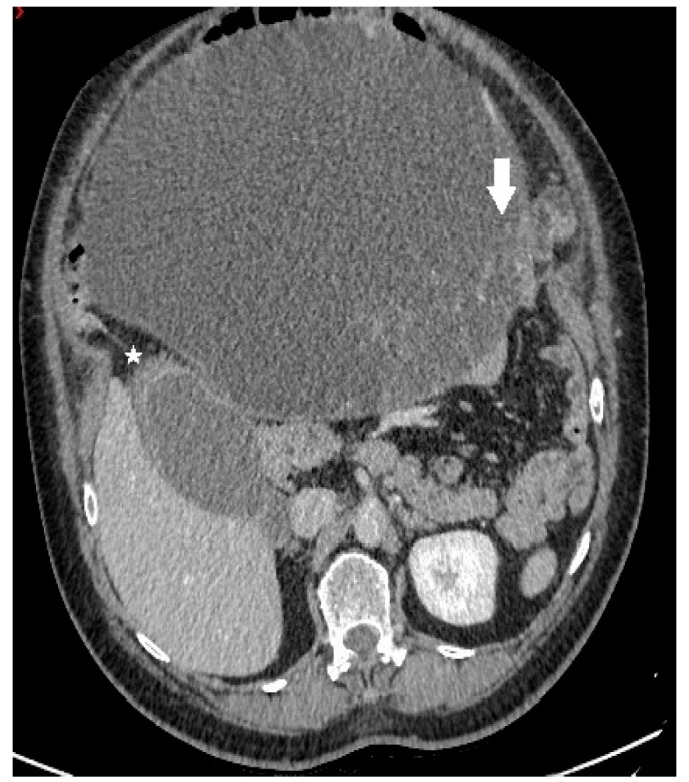
Axial contrast-enhanced CT image demonstrates a predominantly cystic mass, with enhancing papillary projections (white arrow) arising from the left adnexa (not shown). Notice markedly thickened gallbladder wall with alternating areas of high (white star) and low attenuation, giving a striated appearance with mucosal defect—CT signs of acute gangrenous cholecystitis.

**Figure 2:**
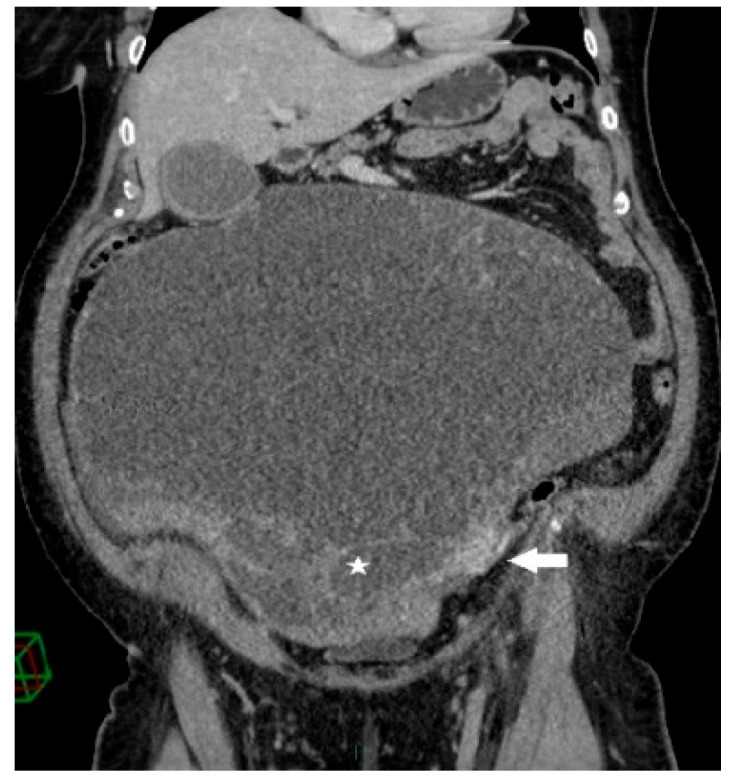
Coronal contrast-enhanced CT image shows a giant predominantly cystic mass with innumerable tiny locules (white star) of variable attenuation arising from the left ovary, which is surrounded by dilated periovarian veins (white arrow).

**Figure 3:**
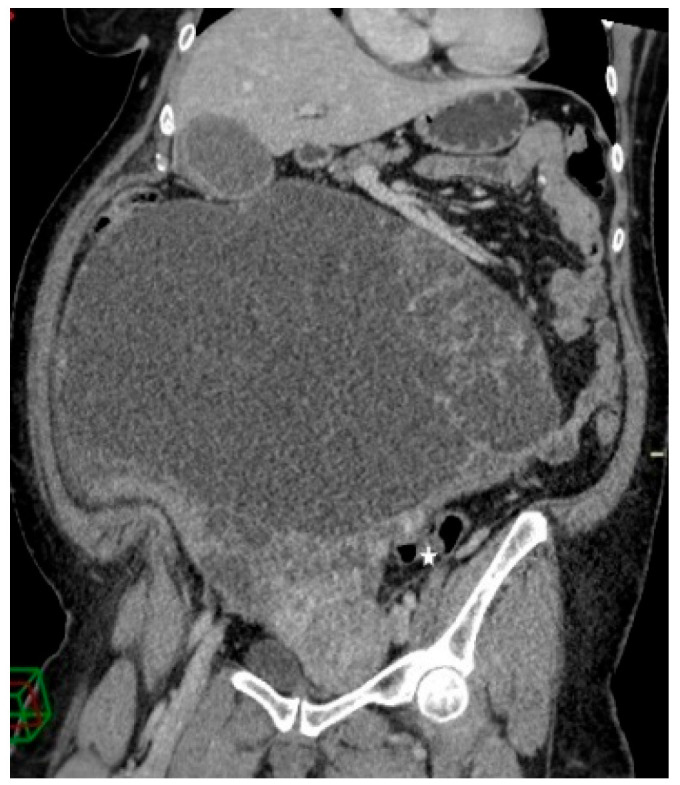
Contrast-enhanced abdominopelvic CT—curved reformation shows a large abdominopelvic cystic multiseptated mass, with a mass effect on the sigmoid colon (white star).

A 68-year-old female patient was admitted to our department due to increasing symptoms of abdominal bloating over the past four months. Other symptoms associated with ovarian cancer were denied by the patient. The patient’s medical history included surgery due to a rupture of an ovarian cyst, while a right adnexectomy was performed 43 years ago. Further, she had hypertension and glaucoma after antihypertensive therapy (Presolol, Asupt, Xalacon). Preoperative CT examination of the abdomen and pelvis confirmed the presence of a tumor in the abdomen and pelvis with a diameter of 335 mm × 275 mm × 345 mm. According to the CT features, the tumor was displacing the surrounding structures, albeit without signs of their infiltration. Initially, the tumor corresponded to left ovarian cystadenocarcinoma (Figure 1, Figure 2 and Figure 3). No intraluminal pathological changes were detected during upper flexible endoscopy and colonoscopy.

During the period one month before surgery values of tumor markers increase from CA 125 82.5 and Ca 19-9 398 to CA 125 200 and CA 19-9 1080 (reference range (RR) CA 125 0–35, CA 19–9 0–37).

**Figure 4:**
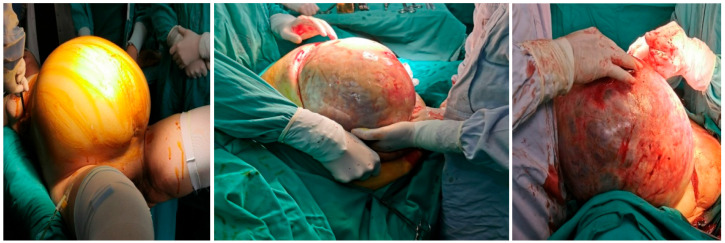
Intraoperative view.

Preoperative blood analysis (BA) showed increased values of white blood cells (WBC) 12.3 × 10^9^/L (CRP 136 mg/L (normally 0–8 mg/L) two days before surgery as part of preoperative preparation, with no other significant abnormalities in BA and biochemistry. After adequate preoperative preparation with the permission of the anesthesiologist, the patient underwent surgery that included excision of the described tumor lesions, hysterectomy with left adnexectomy, cholecystectomy, total omentectomy with peritonectomy of the pelvis and paracolic spaces on both sides, and appendectomy. Intraoperatively, a gangrenous gallbladder without perforation was detected. This was consistent with the preoperative CT findings, and so cholecystectomy was performed. At the end of the operation, we administrated HIPEC (cisplatin 78 mg, doxorubicin 18 mg) via a hyperthermic Belmonth pump for 60 min. The intraoperative view is presented in Figure 4.

On the postoperative day, WBC was 23.2 × 10^9^/L; this did not increase until the fourth postoperative day. The WBC level was increased to 43.9 × 10^9^/L on the 5th postoperative day, with a tendency for further growth. This reached a maximum on the 8th postoperative day with maximum values of 83 × 10^9^/L without an increase in other parameters of inflammation. CRP on the first postoperative day was at a level of 141 mg/L, with a progressive decrease. On the 8th postoperative day, when values of leukocytes were the highest, the value of CRP was 52.7 mg/L, and PCT (procalcitonin) was 0.72 ng/mL (RR < 0.5 ng/mL). The highest value of PCT was seen on the 7th postoperative day, without significant changes in other parameters of BA (hemoglobin and platelets were without significant changes). The leukocyte count with dominant neutrophils was 79.60% preoperatively (postoperatively in the range of 91.00–93.70%) (RR 40–70%), and lymphocytes were at a level of 9.00% preoperatively (postoperatively 4.10–2.66%) (RR 20–50%). Serum electrolyte examination showed hypercalcemia (preoperatively calcium was 2.75 mmol/L, was 2.93 mmol/L on the 8th postoperative day, and on the 9th postoperative day reached 3.15 mmol/L (RR 2.15–2.65 mmol/L). Phosphate levels were within the reference range. The coagulation profile and liver enzymes (AST, ALT, alkaline phosphatase, gamma-glutamyl trasferase, serum bilirubin) in the postoperative period were within the reference range. Other blood tests; pancreatic enzymes (amylase and lipase) and renal function (urea and creatinine) were in RR during hospitalization.

**Figure 5:**
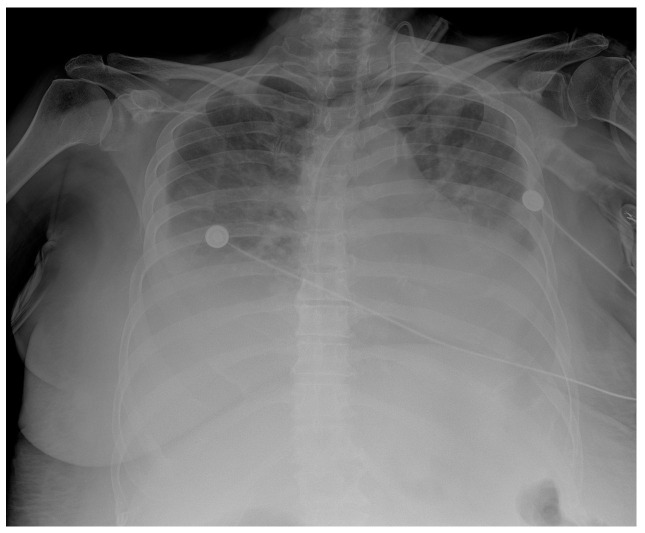
Chest X-ray chest description—A CVC is inserted via UV in the correct position into the SVC. Upper and middle lung zones bilaterally have diffusely decreased transparency, with small discreet peripheral patchy areas of suspected consolidation COVID infection, correlating with clinical and laboratory results. Lower lung zones are bilaterally covered with a pleural effusion that fills costophrenic sinuses. The cardiac shadow is enlarged.

In addition, a postoperative CT scan of the chest, abdomen, and pelvis was performed. This revealed multiple focal changes in the lung, 5 to 10 mm in diameter, and a micronodular lesion of metal intensity approximately 4 mm in diameter in the middle lobe of the right lung. After the hematologist’s consultation, a bone marrow biopsy and bone marrow impression were performed. The findings of the bone marrow biopsy showed the presence of reactive bone marrow without elements of a hematologic diagnosis. Zolendronate was introduced to therapy on the 8th postoperative day by a hematologist. Respiratory support via high-flow nasal cannulae with vasopressor support using norepinephrine was initiated in the patient due to respiratory deterioration on the 9th postoperative day. The patient’s respiratory function and general condition continued to deteriorate and became fatal on the 11th postoperative day. Repeated rapid antigen and PCR tests for COVID were not positive, and the chest radiograph showed consolidation with suspected infection of SARS (Figure 5).

Definitive pathohistological and immunohistochemical findings were in favor of a cystadenocarcinoma mucinous ovary. We found carcinoma anaplastic sarcomatoid ovarii and disseminated peritnei. A poorly differentiated (anaplastic) component in the mural portion of a mucinous ovarian tumor corresponds to anaplastic sarcomatoid carcinoma. Given the presence of multiple infiltrates and vaguely circumscribed flattened nodules, being located not only in the subperitoneal portion but also in the deep part of the ovarian tumor, this could also represent the presence of anaplastic carcinomas, as sometimes seen in mucinous cystadenocarcinomas. That is, it could be the equivalent of the so-called multiple “wall nodules”. Such a pathohistological finding could correspond to the so-called “anaplastic carcinoma arising in mucinous cystadenocarcinoma of the ovary”, possibly of the rhabdoid/spindle type with a disease stage of FIGO IIIC.

In terms of immunophenotype, the anaplastic component of the tumor infiltrate in the ovary showed marked immunoreactivity for vimentin (+++) and pan-cytokeratin (+++), and a scattered portion of tumor cells showed EMA (+), as well as WT-1 (++) and, very rarely, focal change and thrombomodulin (+/−). IHH was performed twice for calretinin and showed no immunoreactivity, nor was any seen for CK5, D2-40, desmin, S-100 protein, LCA, and synaptophysin. Spindle-shaped, desmin-positive cells in the vimentin-positive tumor stroma were seen in places around large anaplastic cells. 

**Figure 6:**
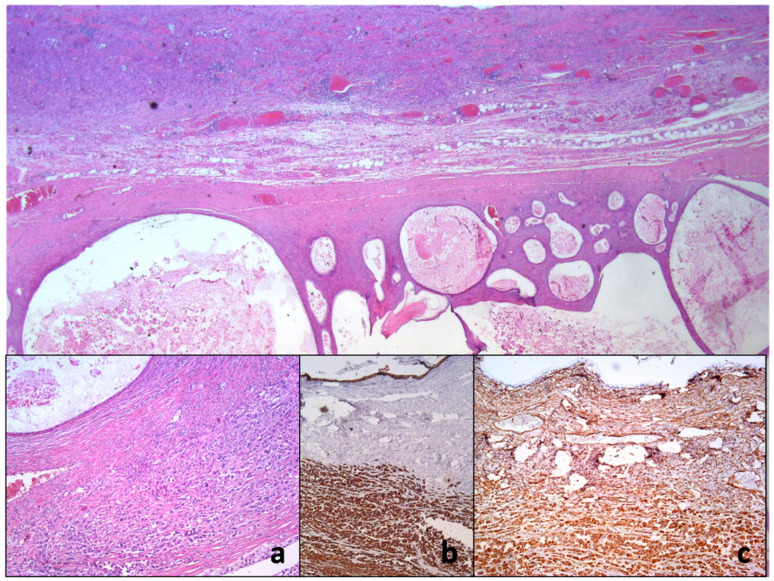
Histopathology of ovarian mucinous cystadenocarcinoma with anaplastic mural node reveals collision of two neoplastic proliferation. On closer inspection, there is anaplastic cancerous infiltration nearby the mucin-rich neoplastic cystic epithelium (**a**). Immunohistochemical examination shows only vimentin (**b**) and pan-cytokeratin immunoexpression using AE1/AE3 monoclonal antibody (**c**).

The cytologic category was positive with the presence of large malignant cells, but in such a way that it was not possible to distinguish with certainty whether the tumor was primary or metastatic (Figure 6).

## 3. Discussion

In the long history of nearly three decades of cytoreductive surgery and HIPEC in the treatment of advanced ovarian cancer, colon cancer, and malignant peritoneal mesothelioma at our department, the occurrence of extreme leukocytosis has not yet been observed after the described procedure. 

According to our knowledge, there is no case in the literature to this date in which the use of cytoreductive surgery and HIPEC in the treatment of mucinous ovarian cancer is associated with postoperative extreme leukocytosis, nor is there an association of acute cholecystitis with MOC.

Cytoreductive surgery combined with HIPEC is associated with a postoperative morbidity of 25–51% and a postoperative mortality of 0–18% [16].

The inflammatory cascade after surgery is associated with granulocytosis (neutrophilia and leukocytosis), the effects of which are conditioned by the increase in proliferation of the granulocyte lineage, the shortening of the time required for granulocyte precursor maturation, and the release of mature cells from the reservoir of bone sinusoids [17]. The mobilization of granulocytes from the bone marrow is the major cause of leukocytosis during stress and systemic inflammatory responses [17].

Hematologic toxicity is a complication that has not been well studied due to the small number of studies that have been performed on this topic. Additionally, the results on this issue are highly variable, with an incidence of 2–38% [16]. There are studies suggesting that preoperative leukocytosis in epithelial ovarian cancer is associated with a more malignant phenotype of the disease and a worse prognosis. In the study by Chen et al. that investigated this association, preoperative leukocyte levels had the highest value of 19.8 × 10^9^/L [18], whereas in our case the preoperative value was 12.3 × 10^9^/L, although the presence of radiographically described cholecystitis must also be taken into account. In the study by Pintado et al., some form of hematologic complication occurred in 77.1% of cases, leukopenia occurred in 8.3%, anemia was seen in 66.7%, and 22.9% patients had coagulopathy [16]. Leukopenia was seen in 22.2% of patients with ovarian cancer and in patients who received HIPEC (doxorubicin, cisplatin) [16]. 

Compared to other studies, the study by Poucke et al. described the absence of postoperative leukopenia, but without extreme values of WBC. The white blood cell count increased significantly from the start of surgical incision to the application of chemotherapy, and the increased leukocyte count compared with the start of surgery was maintained in the first seven postoperative days, although there was no difference in patients with and without splenectomy. The average value of leukocytes on the first postoperative day was 10.5 × 10^9^/L, it was 6.4 × 10^9^/L on the 3rd postoperative day, while it was 8.08 × 10^9^/L on the 7th postoperative day. According to their study, the highest leukocyte count measured was 12.5 × 10^9^/L on the first postoperative day [19].

In a study by Granger et al. [11] into 758 patients with extreme leukocytosis (>40,000), hematopoietic growth factor was associated with 69% of cases, and 15% of patients had infection. High doses of corticosteroids and/or vasopressin were present in 5% of cases, newly diagnosed leukemia in 9%, and PLR in 10% [11]. Pneumonia was present in 49% of patients with proven infection, positive blood culture in 27% of cases, urinary tract infection in 16%, peritonitis in 13%, and wound infection in 13%. Septic shock occurred with a negative blood culture in 3% of patients. Colitis was present in 1% of cases and meningitis was seen in 1% of cases [11]. Among patients with PLR, almost all had a large tumor mass or widespread metastases, which were present in 78% of cases, while pulmonary metastases were detected in 53% of cases. The mean leukocyte count in a patient with PLR was 53 × 10^9^/L (40.6–115.1 × 10^9^/L), with neutrophilia predominating in 96% [11]. According to the results of the study by Granger et al., the majority of patients with PLR were afebrile, 93% were normotensive, 88% had a normal respiratory function, while tachycardia was present in 53%. The prognosis of patients was poor: 76% of patients died within the first 12 weeks after the first leukocytosis, and only 8% of patients survived for more than one year after the first leukocytosis [11].

A review of the literature also revealed that some tumors that secrete G-CSF simultaneously secrete PTHrP (parathyroid hormone-related protein), resulting in hypercalcemia. This hypercalcemia-hyperleukocytosis syndrome is commonly described in neck and head cancers [20]. In a study that included 225 oral cavity carcinomas, 4.4% had hypercalcemia, 4.9% had leukocytosis, and 2.2% had combined hypercalcemia and leukocytosis [21]. Hypercalcemia in these patients ranges from 6.6–8.5 mEg/L, whereas in our case the concentration increased to 3.1 mmol/L [21]. Other tumor markers associated with these tumors include lactate dehydrogenase (LDH), CRP, carcinoembryonic antigen (CEA), and CA 19–9 [20]. In our case, LDH was elevated in the postoperative period, with the highest value of 536.

The presence of PLR in undifferentiated ovarian carcinoma has also been described, although this is without any information on the histological subtype of ovarian carcinoma and the form of treatment [20].

Extreme leukocytosis is usually not associated with infection [11], which was also demonstrated in our case after radiological diagnosis and a decrease in inflammatory markers during the postoperative period.

PLR should be considered as a differential diagnosis in clinically stable patients with high tumor burden [11].

The presence of a leukemoid reaction in a COVID-19 patient with a severe clinical picture has been described in several cases, with a leukocyte range of 70.9–96.6 × 10^9^/L and with a fatal outcome in the period of 5–14 days from hospitalization [22].

## 4. Conclusions

The patient’s death prevented us from performing further diagnostic work into the presence of distant bone metastases, which could not be confirmed via preoperative diagnostics. Due to insufficient literature data, it is difficult to completely understand the clinical picture and prognosis in such cases. Doing so will require the further collection and analysis of data related to this topic with the aim of defining clear diagnostic and therapeutic guidelines.

## Data Availability

Not applicable.
